# *Drosophila* myosin mutants model the disparate severity of type 1 and type 2B distal arthrogryposis and indicate an enhanced actin affinity mechanism

**DOI:** 10.1186/s13395-020-00241-6

**Published:** 2020-08-15

**Authors:** Yiming Guo, William A. Kronert, Karen H. Hsu, Alice Huang, Floyd Sarsoza, Kaylyn M. Bell, Jennifer A. Suggs, Douglas M. Swank, Sanford I. Bernstein

**Affiliations:** 1 Department of Biology, Molecular Biology Institute and Heart Institute, San Diego State University, San Diego, CA 92182-4614 USA; 2 Department of Biological Sciences & Biomedical Engineering, Center for Biotechnology and Interdisciplinary Studies, Rensselaer Polytechnic Institute, Troy, NY 12180 USA

**Keywords:** Distal arthrogryposis, *Drosophila melanogaster*, Myopathy, Myosin, Skeletal muscle

## Abstract

**Background:**

Distal arthrogryposis (DA) is a group of autosomal dominant skeletal muscle diseases characterized by congenital contractures of distal limb joints. The most common cause of DA is a mutation of the embryonic myosin heavy chain gene, *MYH3*. Human phenotypes of DA are divided into the weakest form–DA1, a moderately severe form–DA2B (Sheldon-Hall Syndrome), and a severe DA disorder–DA2A (Freeman-Sheldon Syndrome). As models of DA1 and DA2B do not exist, their disease mechanisms are poorly understood.

**Methods:**

We produced the first models of myosin-based DA1 (F437I) and DA2B (A234T) using transgenic *Drosophila melanogaster* and performed an integrative analysis of the effects of the mutations. Assessments included lifespan, locomotion, ultrastructural analysis, muscle mechanics, ATPase activity, in vitro motility, and protein modeling.

**Results:**

We observed significant defects in DA1 and DA2B *Drosophila* flight and jump ability, as well as myofibril assembly and stability, with homozygotes displaying more severe phenotypes than heterozygotes. Notably, DA2B flies showed dramatically stronger phenotypic defects compared to DA1 flies, mirroring the human condition. Mechanical studies of indirect flight muscle fibers from DA1 heterozygotes revealed reduced power output along with increased stiffness and force production, compared to wild-type controls. Further, isolated DA1 myosin showed significantly reduced myosin ATPase activity and in vitro actin filament motility. These data in conjunction with our sinusoidal analysis of fibers suggest prolonged myosin binding to actin and a slowed step associated with Pi release and/or the power stroke. Our results are supported by molecular modeling studies, which indicate that the F437I and A234T mutations affect specific amino acid residue interactions within the myosin motor domain that may alter interaction with actin and nucleotide.

**Conclusions:**

The allele-specific ultrastructural and locomotory defects in our *Drosophila* DA1 and DA2B models are concordant with the differential severity of the human diseases. Further, the mechanical and biochemical defects engendered by the DA1 mutation reveal that power production, fiber stiffness, and nucleotide handling are aberrant in F437I muscle and myosin. The defects observed in our DA1 and DA2B *Drosophila* models provide insight into DA phenotypes in humans, suggesting that contractures arise from prolonged actomyosin interactions.

## Background

Distal arthrogryposis (DA) is a group of autosomal dominant skeletal muscle disorders characterized by non-progressive congenital contractures in at least two body sites, primarily the upper and lower limbs, which result in camptodactyly and clubfeet [1–3]. In addition to these common manifestations, facial abnormalities frequently occur. There are ten DA syndromes, which are classified according to contracture severity [1, 4, 5]. Three forms (DA2A, DA2B, DA1) are often caused by dominant mutations in the embryonic myosin heavy chain gene, *MYH3* [4]*.* DA2A or Freeman-Sheldon Syndrome (OMIM 193700 http://www.omim.org) is a severe form of the disease characterized by clubfeet, clenched fists, scoliosis, and distinctive facial abnormalities, including a very small mouth, pinched lips, and H-shaped dimpling of the chin (DA2A was recently suggested to be renamed to Freeman-Burian Syndrome [6]). DA2B or Sheldon-Hall Syndrome (OMIM 601680 http://www.omim.org) is a common type of DA that is somewhat less severe than DA2A. In addition to congenital contractures, DA2B is characterized by a distinctive face with a down-slanting palpebral fissure and a small mouth. DA1 (OMIM 108120 http://www.omim.org) is the mildest type of DA. Patients exhibit mild contractures and lack facial dysmorphism.

Analysis of the mutant *MYH3*-induced defects in developing DA muscle is challenging, as MYH3 production is largely restricted to the embryonic period prior to birth (from 6 to 24 weeks of fetal development), and expression is eliminated by the 37th week of gestation [7, 8]. However, expression of *MYH3* occurs during muscle regeneration and has been detected in some adult muscles [9–11]. This has permitted mechanical assays to be performed on adult DA2A-containing muscle cells, which showed that decreased cross-bridge detachment kinetics may explain the congenital contractures observed in this syndrome [10]. In addition, transient kinetics documented ATPase defects of human DA2A myosins expressed in vitro [12]. Further, *Drosophila* models of DA2A display myofibril disarray that likely arises from reduced ATPase activity and reductions in the ability to induce actin sliding [13, 14].

In contrast to the abovementioned insights into the disease mechanism for *MYH3*-based DA2A, no disease models, muscle fiber studies, or biochemical assays have been reported for DA1 or DA2B. Here, we describe the production and analysis of *Drosophila* models of these diseases, with mutations in their muscle myosin motor domains. These models are advantageous in permitting exploration of the homozygous and heterozygous states, assessment of myofibril assembly, degeneration, and function, as well as allowing the preparation of pure mutant myosin for in vitro analysis. Examination of the homozygous state permits insight into the defects directly imparted by the mutant myosin, whereas analysis of the heterozygous state permits an understanding of the dominant basis of the human disease conditions. Our results demonstrate that the severity of the mutant model phenotypes mirror the human condition, i.e., the A234T DA2B mutation is dramatically more severe than the F437I DA1 mutation with regard to longevity, abnormal development, and degeneration of muscle structure, as well as muscle dysfunction. Despite its milder phenotype, *F437I*/+ muscle fibers show reductions in power production and ATP affinity as well as slowed fiber kinetics, increased tension, and enhanced stiffness in mechanical assays. Further, F437I myosin displays reduced ATPase activity and an ~50% reduction in actin filament in vitro sliding velocity compared to wild-type controls. Our results, in conjunction with molecular modeling that documents specific intramolecular interactions affected by each mutation, provide the first insights into the mechanistic basis of these forms of DA.

## Materials and methods

### Molecular cloning of *pwMhcA234T* and *pwMhcF437I*

To produce a subclone of the *Drosophila* myosin heavy chain gene (*Mhc*) required for A234T mutagenesis, plasmids pLitmus and pPA (a *Mhc* subclone) were double digested with Pst I and Bam HI. The resulting 2.8 kb fragment from pLitmus and the 2.1 kb wild-type *Mhc* gene fragment from pPA were gel-isolated and ligated. Using a site-directed mutagenesis kit (QuikChange II Site-Directed Mutagenesis Kit, Agilent Technologies, Santa Clara, CA) and an exon-specific primer pair (forward primer 5′-GCCTTCGGTAAC**A**CCAAGACCGTGCGT-3′, reverse primer 5′-ACGCACGGTCTTGG**T**GTTACCGAAGGC-3′), we created the subclone pLitmusA234T (A234T nucleotide change shown in bold and codon change is underlined). The pLitmusA234T and pPA were then digested with Pst I and Bam HI. The resulting 2.1 kb fragment from pLitmusA234T and the 2.8 kb fragment from pPA were gel-isolated and ligated to form plasmid pPAA234T, carrying 4.3 kb of the *Mhc* gene with the A234T mutation. pPAA234T and pXA, containing an adjacent region of *Mhc*, were then double digested with Pst I and Avr II. The resulting 4.3 kb fragment from pPAA234T and 2.8 kb fragment from pXA were gel-isolated and ligated to form plasmid pXAA234T, carrying 6.8 kb of the *Mhc* gene with mutation A234T. pXAA234T and pwMhc5′ were then digested with Xho I and Avr II. The resulting 6.8 kb fragment from pXAA234T and the 14 kb fragment from pwMhc5′ were gel-isolated and ligated to form pwMhc5′A234T, carrying 19.3 kb of the *Mhc* gene with mutation A234T. pwMhc5′A234T and pwMhc3′ were digested with Eag I. The resulting 19.3 kb fragment from pwMhc5′A234T and the 12.5 kb fragment from pwMhc3′ were gel-isolated and ligated to form a 31.8-kb plasmid, pwMhcA234T, carrying the entire 23.8 kb *Mhc* gene with mutation A234T. After each ligation step, the subclones were sequenced (Eton Bioscience, Inc., San Diego, CA) to ensure the presence of the desired mutation with no unwanted changes. The entire coding region and splice junctions of the final pwMhcA234T plasmid were sequenced before *P* element-mediated transformation.

To produce the subclone for F437I mutagenesis, plasmids pLitmus and pKS (a myosin subclone) were double digested with Nsi I and Bgl II. The resulting 2.8 kb fragment from pLitmus and the 1.4 kb fragment from pKS were gel-isolated and ligated to form a plasmid carrying 1.4 kb of the wild-type *Mhc* gene. We used site-directed mutagenesis with an exon-specific primer pair (forward primer 5′-TTCGATCGTCTG**A**TCAAGTGGCTGGTG-3′ and reverse primer 5′-CACCAGCCACTTGA**T**CAGACGATCGAA-3′), to create subclone pLitmusF437I (F437I nucleotide change is shown in bold and codon change is underlined), carrying 1.4 kb of the *Mhc* gene with the F437I mutation. pLitmusF437I and pKS were then digested with Nsi I and Bgl II. The resulting 1.4 kb fragment from pLitmusF437I and the 2.8 kb fragment from pKS were gel-isolated and ligated to form plasmid pKSF437I, carrying 2.4 kb of the *Mhc* gene with the F437I mutation. The pKSF437I and pwMhc5′ plasmids were then digested with Avr II and Sph I. The resulting 2.4 kb fragment from pKSF437I and 18 kb fragment from pwMhc5′ were gel-isolated and ligated to form pwMhc5′F437I, carrying 19.3 kb of the *Mhc* gene with mutation F437I. pwMhc5′F437I and pwMhc3′ were then digested with Eag I. The resulting 19.3 kb fragment from pwMhc5′F437I and 12.5 kb fragment from pwMhc3′ were gel-isolated and ligated to form the 31.8 kb plasmid pwMhcF437I, carrying the entire 23.8 kb *Mhc* gene with mutation F437I. The sequencing of the intermediate and final products was carried out as described above.

### *P* element transformation of *Mhc* genes

The plasmids carrying *P* transposable elements [15] were injected by BestGene, Inc. (Chino Hills, CA) into *Drosophila melanogaster* embryos in the presence of transposase to produce transgenic lines. Approximately 2400 and 1200 embryos were injected with pwMhcA234T and pwMhcF437I, respectively. Balancer chromosomes and standard genetic crosses were utilized to map transgene locations. For pwMhcA234T, eleven independent lines were obtained. Three mapped to the X chromosome, three to the second chromosome, and five to the third chromosome. For pwMhcF437I, sixteen independent lines were obtained. Three mapped to the X chromosome, seven to the second chromosome, and six to the third chromosome. For each construct, two independent inserts located on the third chromosome were crossed into the *Mhc*^*10*^ IFM and jump muscle myosin null background [16], yielding lines *A234T-2*, *A234T-4*, *F437I-3*, and *F437I-4*.

### Reverse transcription and polymerase chain reaction (RT-PCR)

RNA was extracted from 20 to 25 dissected upper thoraces of *Drosophila* using the LiCl extraction method [17]. cDNA was synthesized using the Protoscript cDNA synthesis RT-PCR kit (New England Biolabs, Ipswich, MA) along with 3 μl of 0.2 μg/μl of a specific reverse primer listed below. PCR was performed using 1 μl of cDNA and 2 μl of forward and reverse primers. For F437I, the forward primer (5′-CGATACCGCCGAGCTGTACAG-3′) and reverse primer (5′-GAGCTTCTTGAAGCCCTTACGG-3′) were employed to amplify exon 8 through exon 12. For A234T, the forward primer (5′-TGGATCCCCGACGAGAAGGA-3′) and reverse primer (5′-TACGGCCCTGGGTGACGAAC-3′) were used to amplify exon 2 through exon 8. PCR conditions were generally set at 3 min at 94 °C, followed by 34 cycles of 30 s at 94 °C, 30 s at 60 °C, and 90 s at 68 °C, followed by 5 min at 68 °C. RT-PCR products were sequenced by Eton Bioscience, Inc. (San Diego, CA). In addition, primers adjacent to alternative exons were used to sequence cDNA from both mutants to assess motor domain alternative exon splicing patterns. Reverse primer (5′-CAGAGATGGCGAAAATATGG-3′) revealed exon 3b was used. Forward primer (5′-AAAGACTGAGAACACCAAGA-3′) and an additional forward primer (5′-GGCTGGTGCTGATATTGAGA-3′) revealed exon 7d was used. Reverse primer (5′-GAACATAGACTCTTCCTCCAGG-3′) revealed exon 9a was used. Forward primer (5′-GTTCCCCAAGGCCTCCGATCA-3′) revealed exon 11e was used.

### Myosin expression levels

Protein accumulation was determined by SDS-polyacrylamide gel electrophoresis [18]. Six dissected upper thoraces from adult flies were homogenized in 180 μl of Laemmeli loading buffer containing β-mercaptoethanol. Samples were loaded on Mini-PROTEAN TGX precast gels (Bio-Rad, Hercules, CA). Gels were stained with GelCode Blue Stain Reagent (ThermoFisher Scientific, Carlsbad, CA) and then were digitally scanned using an Epson Perfection 1640SU flatbed scanner. Each transgenic line was tested three times with three different individual samples. Quantification of band intensity was performed using UN-SCAN-IT gel 6.1 software. The myosin to actin ratios of transgenic flies were calculated and compared to the ratio for *pwMhc2* control flies of the same age, with statistical differences tested by one-way ANOVA, with *P* < 0.05 considered significant.

### Viability assay

The lifespans of female homozygotes and heterozygotes for each transgenic fly line and for control flies were tested after placement into vials containing standard fly food, and flies were transferred into fresh vials every 3 days. More than 50 flies from each line were tested, except for homozygous *A234T-2* (14 flies) and *A234T-4* (31 flies), which eclosed at very low rates. The number of surviving flies was recorded every other day.

### Flight and jump ability

Flight ability of female homozygotes and heterozygotes for each transgenic fly line and for control flies was tested at room temperature at 2 days or 2 weeks post eclosion. At least 50 flies were assayed per genotype by release into a Plexiglas box with a light at its top to elicit a phototropic response [19]. The flight index was calculated using the formula 6 *U*/*T* + 4*H*/*T* + 2*D*/*T* + 0 *N*/*T*, where *U* is the number of flies flying upward, *H* is the number flying horizontally, *D* is the number flying down, *N* is the number of flightless flies, and *T* is the total number of flies tested [20]. Flight indices were calculated using cohorts of ~ 15 flies. Wing beat frequency was assessed using nylon-tethered flies and an optical tachometer. Three flights of 20 s duration were recorded and the average frequency of the three reported [20]. The jump ability of female homozygotes and heterozygotes for each transgenic fly line and for control flies was tested at room temperature. CO_2_ anesthesia was administered and wings were cut on the first day post-eclosion. Jump ability was tested the following day, with more than 20 flies tested from each fly line. Jump distances were assessed 10 times and the top 3 distances were averaged to yield the final value [21]. One-way ANOVA and Kruskal-Wallis tests were performed for flight indices and for jump abilities (GraphPad Prism, La Jolla, CA) and differences between two groups were considered significant at *P* < 0.05.

### Electron microscopy

Sample fixation was performed according to an established protocol [22]. Briefly, dorsolongitudinal IFMs were dissected from upper thoraces and placed into primary fixative (3% paraformaldehyde, 2% glutaraldehyde, 100 mM sucrose, 100 mM sodium phosphate buffer, 2 mM EGTA, pH 7.2) at 4 °C overnight. Samples were washed five times with 100 mM sodium phosphate buffer, pH 7.2 and treated with secondary fixative (1% osmium, 100 mM sodium phosphate, pH 7.2) at 4 °C for 2 h. Samples were washed with deionized water six times and dehydrated by acetone treatment at room temperature. After embedding and overnight resin polymerization, samples were sectioned on an ultramicrotome. Thin sections were stained with uranyl acetate followed by lead citrate and examined on a FEC Tecnai 12 transmission electron microscope.

### Fiber mechanical studies

Isolation, preparation, and mechanical analyses of IFM of 2-day-old control and mutant flies were performed as described previously [23]. In brief, skinned IFM fibers were attached to aluminum T-clips, which were used to mount individual fibers onto a temperature-controlled (15 °C) muscle mechanics apparatus. The optimal fiber length was determined by stretching the fiber in 2% increments until maximum power was generated, as measured using sinusoidal analysis [23, 24]. Work loops and ATP response assays were performed on the fiber at this optimal length [23]. Student’s *t* tests were used for statistical comparisons, with *P* < 0.05 considered significant.

### Myosin and actin isolation, myosin ATPase assay, and in vitro motility

Protein isolation, ATPase assays, and in vitro motility procedures have been described in detail previously [25–27]. Briefly, myosin was isolated from dissected dorsolongitudinal IFMs of ~ 150 young flies, and myosin concentration was determined by spectrophotometry. Actin was isolated from frozen chicken breast. ATPase activities of myosin at 2.0 μg/μl were determined in the presence of Mg^2+^ and [γ-^32^P]-ATP. Increasing concentrations of filamentous actin permitted calculation of actin-stimulated ATPase activity (*V*_*max*_) and actin affinity relative to ATPase (*K*_*m*_). This was accomplished by subtraction of basal Mg-ATPase activity and fitting data points to a curve derived from the Michaelis–Menten equation. In vitro motility assays were carried out with myosin at 0.5 μg/μl coated onto a nitrocellulose-treated coverslip. In the presence of ATP, the movement of filamentous actin labeled with fluorescent phalloidin was captured by high-speed video imaging and the sliding velocity of smoothly moving actin filaments was calculated computationally. Mean values from five independent experiments (two technical replicates for each sample) for the ATPase assays or from seven independent motility assays (26-108 filaments/assay) were compared for statistically significant differences (*P* < 0.05) relative to control myosin values using a Student’s *t* test.

### Protein structure analysis

To determine potential intramolecular interactions in wild-type or mutant myosin molecules, the SWISS-MODEL program was employed (http://swissmodel.expasy.org, 2010). The *Drosophila* indirect flight muscle (IFM) wild-type isoform sequence, as well as isoforms with mutations A234T or F437I, were modeled onto the scallop muscle myosin II structure in the presence of Mg.ADP, in both the pre-power stroke state (PDB 1QVI) and in the actin-detached post-power stroke state (PDB 1KK8). Models were examined to detect amino acid residue interactions with DA residues using the PyMOL program (The PyMOL Molecular Graphics System, Version 1.5.0.4 Schrödinger, LLC), an open-source tool to visualize molecules (www.pymol.org). Interactions in the range of 2.5-4 Å were considered significant for forming hydrogen bonds, salt bridges, or hydrophobic contacts.

## Results

**Fig. 1:**
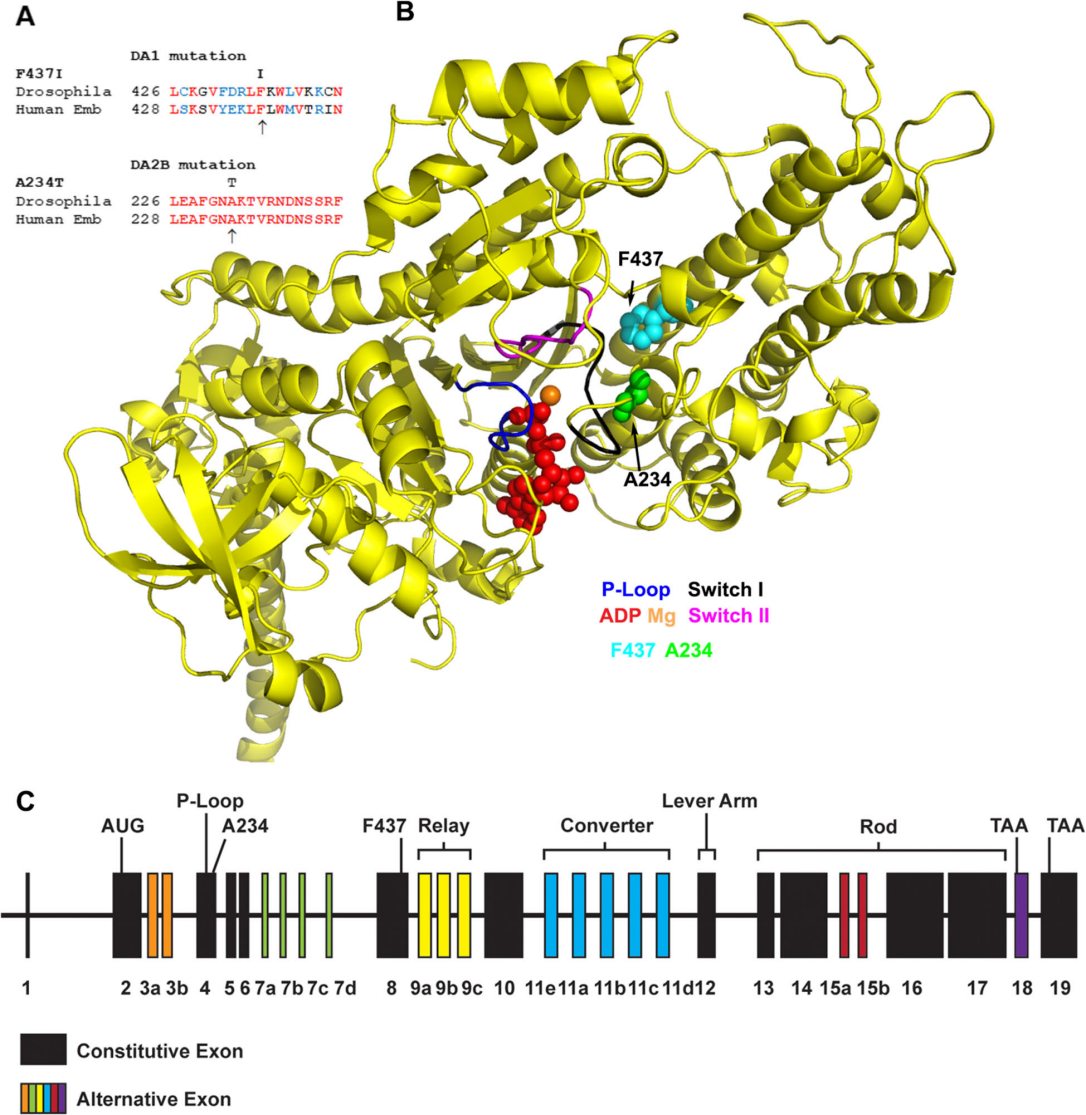
Location of F437 and A234 residues on the myosin molecule. **a** Myosin sequences surrounding the F437 and A234 residues in human embryonic myosin are compared to their *Drosophila* counterparts. Identical residues are shown in red and conserved residues in blue. The mutations that cause DA are shown above the *Drosophila* sequences. **b** Myosin residues F437 (cyan) and A234 (green) of *Drosophila* IFM myosin are modeled on scallop muscle myosin II in the pre-power stroke state (PDB 1QVI) in the presence of the Mg.ADP complex. Functional domains of interest are highlighted: P-loop (blue), switch I (black), switch II (magenta), and Mg.ADP complex (orange, red). F437 and A234 are near to switch I, which is critical for communicating the nucleotide state to the actin-binding site. **c** Map of the *Mhc* transgene, which contains transcriptional enhancers located in the 5′ upstream region and first intron [31], along with the entire genomic sequence through the 3′ end of the gene. Translation start (AUG) and termination (TAA) sites are shown as well as the locations encoding the DA residues and key protein regions

The goal of this study was to produce *Drosophila* models of myosin-based DA1 and DA2B in order to define their phenotypic defects, to examine whether the disparate disease states are mirrored in the disease models and to gain insights into the mechanism of the disease process. We focused on DA1 mutation F437I [28] and DA2B mutation A234T [8]. Both mutant residues are in the myosin motor domain near to switch 1, a nucleotide-sensitive loop that moves from an “open” to “closed” position upon ATP binding, yielding the opening of the actin-binding cleft and facilitating actin release [29, 30]. Based on NCBI BLAST examination, F437 and A234 and their adjacent residues are well conserved in myosins, with 73% conservation between human MYH3 embryonic myosin and *Drosophila* muscle myosin near F437 and 100% identity in the A234 region (Fig. 1a). To localize the DA residues in the *Drosophila* model, the fly indirect flight muscle (IFM) myosin motor domain sequence was modeled onto the scallop muscle myosin II structure in the pre-power stroke state (PDB 1QVI) (Fig. 1b). DA1-related residue F437 (cyan) is located in relatively close proximity to switch I (black) and the ADP molecule (red), whereas DA2B-related residue A234 (green) is located four amino acids upstream of switch I and even closer to the nucleotide.

### Production and verification of F437I and A234T transgenic lines

DNA constructs and transgenic lines carrying the *Mhc* gene with the F437I mutation or the A234T mutation were generated by in vitro mutagenesis and *P* element-mediated germline transformation as detailed in the “Methods” section. The control and mutant transgenes (Fig. 1c) contain the entire *Mhc* gene as well as ~450 bp of the upstream regulatory region along with the first intron, which includes multiple transcriptional enhancers necessary for muscle-specific expression [31]. Two independent lines for each transgene were crossed into the *Mhc*^*10*^ IFM/jump muscle myosin-null background [16]. To verify that the transgenic lines express the expected mutations, RT-PCR was performed. The sequences of the RT-PCR products showed that the F437I mutation is present in exon 8 in lines *F437I-3* and *F437I-4* and that the A234T mutation exists in exon 4 in lines *A234T-2* and *A234T-4*. Sequencing of *Mhc* cDNA from all four transgenic lines verified usage of exons 3b, 7d, 9a, and 11e, indicating that the DA mutations do not affect the *Drosophila* IFM RNA alternative splicing pattern for the motor domain.

To determine relative levels of mutant myosin expression in each homozygous transgenic line, we employed SDS-polyacrylamide gel electrophoresis. We scanned stained gels to determine the level of myosin relative to actin accumulation in upper thoraces. Two-day-old flies from all four lines showed ratios of myosin to actin that were slightly reduced [*F437I-3*: 0.95 ± 0.05; *F437I-4*: 0.93 ± 0.06; *A234T-2*: 0.93 ± 0.09; *A234T-4*: 0.93 ± 0.12], but not statistically different from the normalized *pwMhc2* wild-type transgenic control (1.00 ± 0.03) (one-way ANOVA). The *pwMhc2* transgene has previously been shown to express relative levels of myosin heavy chain that are equivalent to wild type [32].

### Effects of DA1 and DA2B mutations on lifespan

**Fig. 2:**
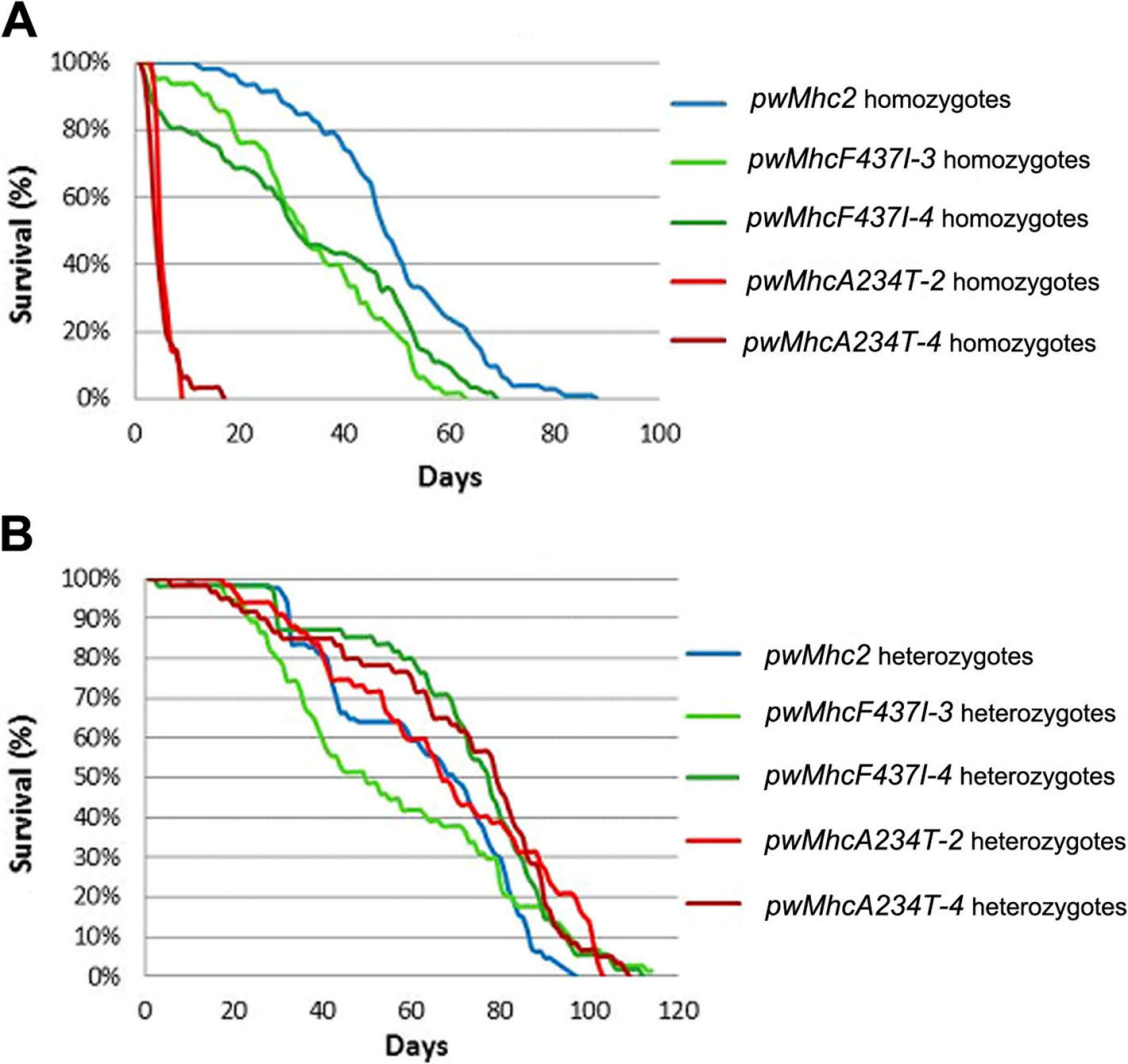
Viability of DA1 and DA2B transgenic female flies. Lifespans of **a** homozygotes and **b** heterozygotes (transgene/+) were determined for *pwMhc2* control, *F437I*, and *A234T* transgenic lines. The percentage of surviving flies from each line was recorded every other day and flies were transferred into fresh vials every 3 days. The major observation is that *A234T* homozygotes are dramatically less viable compared to *F437I* homozygotes and *pwMhc2* controls

We tested homozygous and heterozygous female flies of each fly line to determine how the mutations affect lifespan. Note that the homozygote (two copies transgene) and heterozygote (one transgene and one wild-type gene) designations refer to expression in the IFM and jump muscles, whereas in the muscles that are essential for viability, two (homozygote) or one (heterozygote) copies of the transgene are expressed in a diploid (wild-type) background. In the homozygous group, the half-life of control flies was 48 days. In contrast, the average half-life of *F437I* flies was 32 days and that of *A234T* was 4 days (Fig. 2a). The dramatically decreased lifespan for DA2B homozygotes compared to DA1 homozygotes is consistent with the relative severity of the phenotypes of DA2B and DA1 in the human syndrome. In the heterozygous group (Fig. 2b), DA1 with an average 64-day half-life and DA2B with an average 73-day half-life had similar lifespans compared with the control (70-day half-life), suggesting that the lower mutant gene dosage does not affect longevity.

### Effects of DA1 and DA2B mutations on flight and jump ability

We explored the IFM function of each transgenic line by measuring the ability of homozygotes and heterozygotes to fly up toward a light source (Table 1). *F437I* (DA1) homozygotes, *A234T* (DA2B) homozygotes, and *A234T* heterozygotes were all completely flightless at 2 days post-eclosion. However, 2-day-old *F437I* heterozygotes showed flight ability, although the lines displayed significantly reduced flight indexes of 4.70 ± 0.07 and 4.62 ± 0.04 compared with the control value of 5.51 ± 0.04. This was reflected in decreased wingbeat frequencies for *F437I-4* heterozygotes compared to control, at both room temperature (169.2 ± 2.7 Hz vs. 195.5 ± 2.8 Hz) and 15 °C (134.1 ± 2.1 Hz vs. 156.2 ± 3.0 Hz), respectively (*P* < 0.00001, Student’s *t* test). The flight indexes of *F437I* heterozygotes for 2-week-old flies were 2.90 ± 0.04 and 3.30 ± 0.03 compared to 4.83 ± 0.06 for heterozygous controls (Table 1), indicating an age-dependent differential decrease in flight ability in the mutant heterozygotes.

**Table 1:** Flight and jump ability of DA1 (*F437I*) and DA2B (*A234T*) female transgenic flies

Line name (age)	Flight test, *n*	Up (%)	Horizontal (%)	Down (%)	Not at all (%)	Flight index ± SEM	Jump distance ± SEM
*pwMhc2* (2 days)	122	48.4	29.5	22.1	0	4.54 ± 0.02	7.48 ± 0.20
*F437I-3* (2 days)	106	0	0	0	100	0	2.27 ± 0.08
*F437I-4* (2 days)	100	0	0	0	100	0	2.53 ± 0.13
*A234T-2* (2 days)	50	0	0	0	100	0	0.25 ± 0.02
*A234T-4* (2 days)	50	0	0	0	100	0	0.26 ± 0.01
*pwMhc2*/+ (2 days)	136	79.4	14	6.6	0	5.51 ± 0.04	7.82 ± 0.31
*F437I-3*/+ (2 days)	100	43.0	43.0	14.0	0	4.70 ± 0.07	6.33 ± 0.19
*F437I-4*/+ (2 days)	101	39.6	53.5	6.9	0	4.62 ± 0.04	6.63 ± 0.28
*A234T-2*/+ (2 days)	50	0	0	0	100	0	1.01 ± 0.04
*A234T-4*/+ (2 days)	53	0	0	0	100	0	1.19 ± 0.09
*pwMhc2*/+ (2 weeks)	126	46.8	34.1	19	0	4.83 ± 0.06	–
*F437I-3*/+ (2 weeks )	100	1	41	58	0	2.90 ± 0.04	–
*F437I-4*/+ (2 weeks)	127	3.9	52	44.1	0	3.30 ± 0.03	–

Flight and jump abilities for two independent lines expressing the *F437I* or *A234T* transgene are shown ± standard errors of the mean. Each homozygous transgene stock was in the *Mhc*^*10*^ IFM- and jump muscle myosin-null background [16]. Heterozygotes were generated by crossing these stocks to wild-type *yw* flies. Values are compared to *pwMhc2* (wild-type myosin transgene) control for homozygotes or *pwMhc2*/+, for heterozygotes. For flight testing, cohorts of transgenic flies were assayed for the ability to fly up (U), horizontal (H), down (D), or not at all (N). Flight index and SEM were determined using the mean value of cohorts of ~ 15 flies each, using the following equation: 6*U*/*T* + 4*H*/*T* + 2*D*/*T* + 0*N*/*T*. *T* is the total number of flies tested in a cohort. All flight test values for mutants were significantly less than matched controls (*P* < 0.05). Jump distances were assessed 10 times for each fly, with the three longest distances averaged to yield the final value (> 20 flies/genotype). All jump test values for mutants were significantly less than matched controls (*P* < 0.01)

We examined the function of the jump muscle in homozygotes and heterozygotes at 2 days post eclosion through jump distance assessment. *F437I* and *A234T* transgenic flies displayed statistically significant reductions in jump capabilities relative to controls, for both homozygotes and heterozygotes (Table 1). Heterozygotes exhibited greater jump ability than homozygotes. Furthermore, regardless of whether they were homozygotes or heterozygotes, *F437I* transgenic flies showed less impairment in jump ability compared with *A234T* flies. Overall, the flight and jump muscle function assessments are consistent with the observed human phenotypes, in that DA1 (*F437I*) syndrome is less severe than DA2B (*A234T*).

### DA1 and DA2B mutations cause ultrastructural defects in IFMs of transgenic flies

**Fig. 3:**
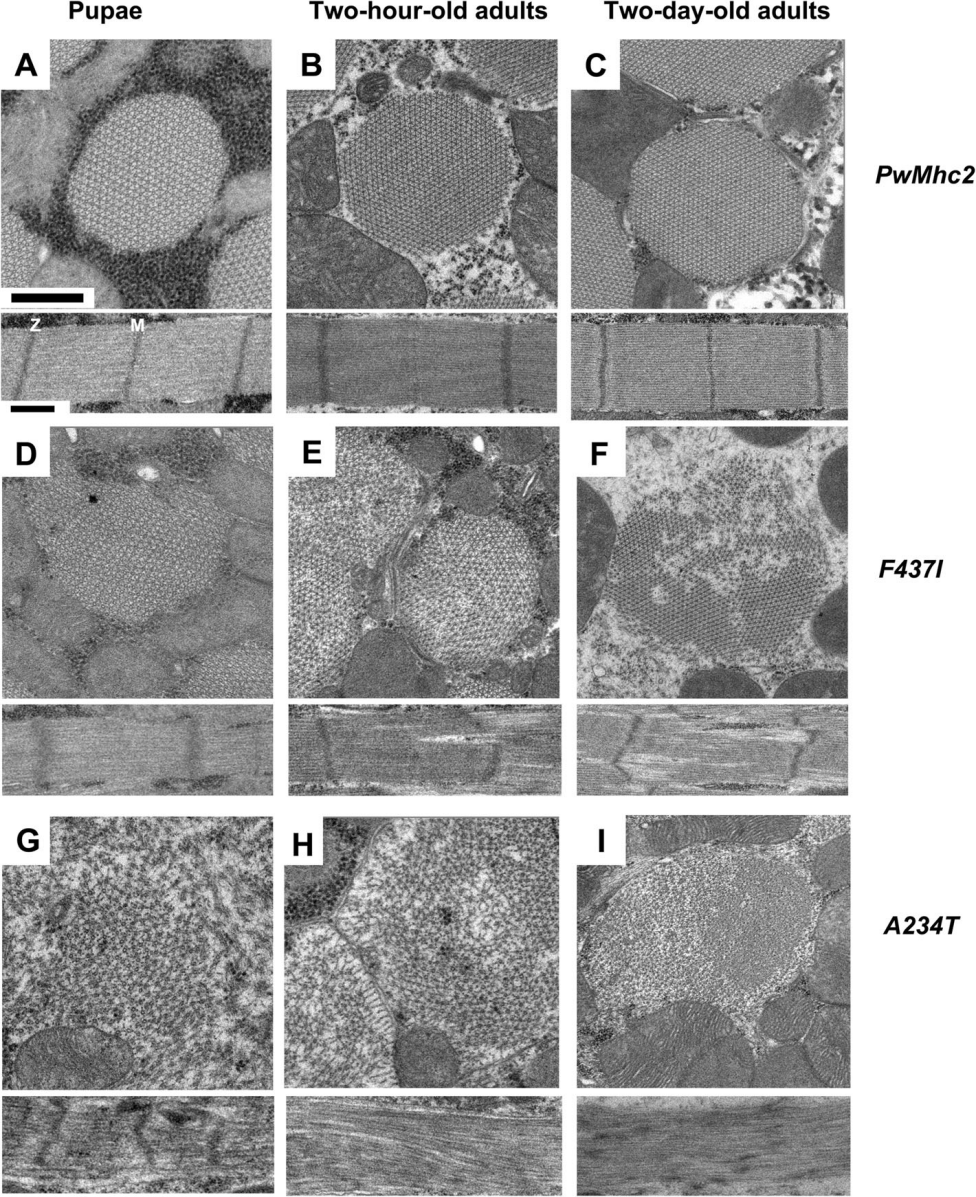
Homozygous *F437I* and *A234T* mutations disrupt myofibril assembly and stability. Each panel is representative of the myofibril population at that given stage of development, although varying levels of degeneration were observed as mutant organisms aged. **a** Transverse and longitudinal sections from wild-type transgenic control (*pwMhc2*) late-stage pupae. Rounded myofibril morphology with normal hexagonal packing of thick and thin filaments is observed, along with clearly demarcated *M*- and *Z*-lines in well-defined sarcomeres. **b** Transverse and longitudinal sections from wild-type transgenic control (*pwMhc2*) 2-h-old adults. Myofibril structure is retained. **c** Transverse and longitudinal sections from wild-type transgenic control (*pwMhc2*) 2-day-old adults. Myofibril structure is maintained. **d** Transverse and longitudinal sections from homozygous *F437I* late-stage pupae. Myofibril morphology and hexagonal packing of thick and thin filaments are abnormal. Sarcomeres display fraying and disrupted *M-* and *Z*-line structure. **e** Transverse and longitudinal sections from homozygous *F437I* 2-h-old adults. Continued disruption in hexagonal packing of thick and thin filaments is observed with more severe sarcomere structural aberrations. **f** Transverse and longitudinal sections from homozygous *F437I* 2-day-old adults. Breakdown in myofibril morphology with thick and thin filament dispersion has occurred. Sarcomeres show granular inclusions. **g** Transverse and longitudinal sections from homozygous *A234T* late-stage pupae. Myofibril morphology and hexagonal packing are poor. Sarcomeres display fraying with abnormal *M*- and *Z*-line structures. **h** Transverse and longitudinal sections from homozygous *A234T* 2-h-old adults. Disruption in myofibril morphology and hexagonal packing of thick and thin filaments continues. The transverse section displays a complete breakdown of sarcomere organization, with skeins of filaments and scattered *Z*-band material. **i** Transverse and longitudinal sections from homozygous *A234T* 2-day-old adults. Disruption in myofibril morphology is severe with scattered thick and thin filaments along with granular material in both transverse and longitudinal sections. M, *M*-line. Z, *Z*-line. Scale bars, 0.5 μm

**Fig. 4:**
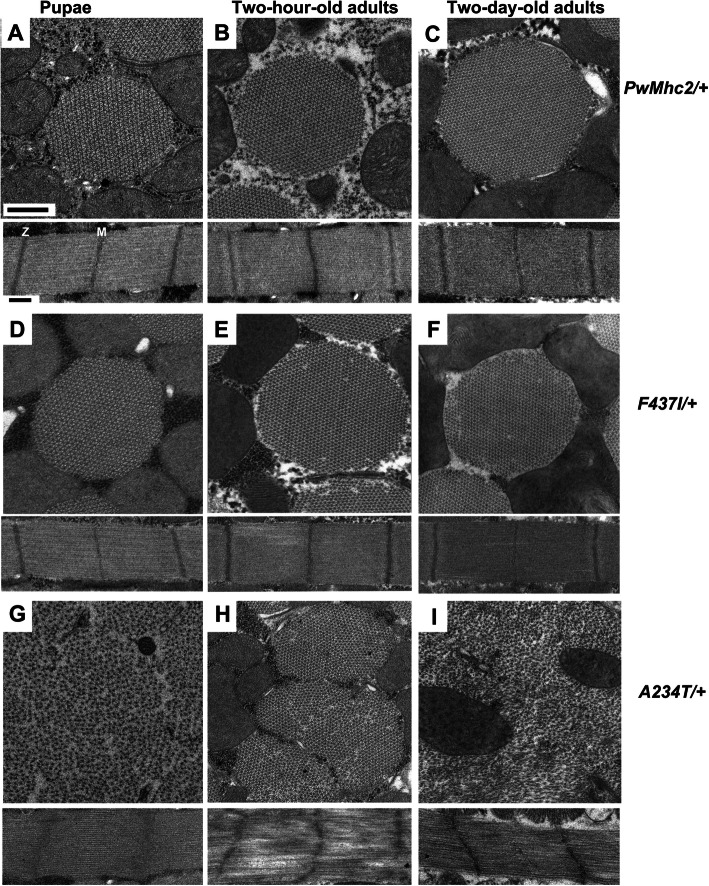
Heterozygous *F437I* and *A234T* mutations differentially affect myofibril assembly and stability. Each panel is representative of the myofibril population at that given stage of development, although varying levels of degeneration were observed as *A234T*/+ organisms aged. **a** Transverse and longitudinal sections from wild-type heterozygous transgenic control (*pwMhc2*/+) late-stage pupae. Rounded myofibril morphology with normal hexagonal packing of thick and thin filaments is observed, along with clearly demarcated *M*- and *Z*-lines in well-defined sarcomeres. **b** Transverse and longitudinal sections from wild-type transgenic control (*pwMhc2*/+) 2-h-old adults. Myofibril structure is retained. **c** Transverse and longitudinal sections from wild-type transgenic control (*pwMhc2*/+) 2-day-old adults. Myofibril structure is retained. **d** Transverse and longitudinal sections from heterozygous *F437I*/+ late-stage pupae. Myofibril morphology, hexagonal packing of thick and thin filaments, and sarcomere structure resemble a wild-type organism (panel **a**). **e** Transverse and longitudinal section from heterozygous *F437I*/+ 2-h-old adults. Aside from occasional missing filaments, leading to mild disruption in hexagonal packing, the sarcomere structure is normal. **f** Transverse and longitudinal sections from heterozygous *F437I*/+ 2-day-old adults. Phenotype is similar to that observed at 2 days. **g** Transverse and longitudinal sections from heterozygous *A234T*/+ late-stage pupae. Myofibril morphology is severely disrupted with some abnormalities in a hexagonal packing. Sarcomeres display *Z*-line irregularities with the absence of *M*-lines. **h** Transverse and longitudinal sections from heterozygous *A234T*/+ 2-h-old adults. Continued disruption in hexagonal packing of thick and thin filaments is observed. *Z*-lines are irregular, with possible sarcomere hypercontraction. **i** Transverse and longitudinal sections from heterozygous *A234T*/+ 2-day-old adults. Severe disruption in hexagonal packing of thick and thin filaments is observed, with a breakdown of myofibril boundaries. Sarcomeres contain skeins of filaments and appear to be hypercontracted. M, *M*-line. Z, *Z*-line. Scale bars, 0.5 μm

To determine if DA1 and DA2B mutations cause ultrastructural defects during myofibril assembly and development, each transgenic line was examined using transmission electron microscopy. To this end, thin sections of homozygotes (Fig. 3) and heterozygotes (Fig. 4) were imaged at the late pupal stage as well as at 2 h and 2 days post-eclosion.

Myofibrils of control *pwMhc2* or *pwMhc2/+* IFMs had similar appearances at each stage of development (Fig. 3a–c and Fig. 4a–c). In transverse sections, myofibrils are circular, with each actin-containing thin filament midway between two myosin-containing hollow thick filaments. Each thick filament is surrounded by six thin filaments, forming a hexagonal lattice. In longitudinal sections, thick and thin filaments are well aligned. Electron dense *Z*-lines clearly define each sarcomere, with each containing a central *M*-line.

IFMs of homozygous *F437I* (DA1) organisms fail to assemble properly and progressively worsen with age. In late-stage *F437I* pupae, myofibrils are distorted with some disruptions of thick and thin filament packing (Fig. 3d). *M*-lines are poorly formed and *Z*-lines are diffuse. In 2-h-old *F437I* adults, more severe filament irregularities are present, with gaps between thick and thin filaments (Fig. 3e). In 2-day-old *F437I* adults, adjacent myofibrils tend to merge (Fig. 3f). Disorganized thick and thin filaments and electron-dense aggregates are scattered throughout the myofibrils. Filament alignment is aberrant.

The assembly of homozygous *A234T* (DA2B) IFM myofibrils is severely disrupted, and this leads to progressive structural degradation. In late-stage pupae, poorly formed myofibrils with abnormal filament packing are evident (Fig. 3g). *M*- and *Z*-lines are extremely distorted. In 2-h-old *A234T* adults, severe defects in packing and alignment of filaments occur, with remnants of *M*- and *Z*-lines diffused throughout the sarcomere (Fig. 3h). In 2-day-old *A234T* adults, thick and thin filaments are dispersed randomly throughout the myofibril along with other electron-dense aggregates, such as *Z*-line material or glycogen granules. In the longitudinal section, filament alignment is highly aberrant and sarcomeres contain scattered electron-dense bodies that may be remnants of *Z*-lines (Fig. 3i).

We next examined the IFMs of heterozygous *F437I* and *A234T* transgenic flies in order to ascertain the dominant phenotypes that are more relevant to the human conditions. *F437I* heterozygous late-stage pupae display normal thick and thin filament packing, along with well-formed sarcomeres (Fig. 4d). For 2-h-old *F437I* heterozygous flies, myofibrils appear essentially normal, with occasional filaments missing from myofibrils that display *M*- and *Z*-lines similar to controls (Fig. 4e). These minor structural defects were carried over into 2-day-old organisms, without obvious worsening of morphology (Fig. 4f).

For late-stage *A234T* heterozygous pupae, myofibril morphology is abnormal, with small myofibrils that show some filament packing disruptions (Fig. 4g). *M*- and *Z*-lines are poorly formed. However, defects are not nearly as severe as for homozygotes (Fig. 3g). For 2-h-old *A234T* heterozygous adults, myofibrils show further disruption in thick and thin filament packing (Fig. 4h). *M*- and *Z*-lines are aberrant or absent. In 2-day-old *A234T* heterozygous adults, some myofibrils appear to have fused (Fig. 4i), with poorly ordered thick and thin filament arrays. *Z*-lines are more closely spaced (~ 2.4 μm) than in control (~ 3.5 μm), suggesting hypercontraction or reduction in filament lengths (Fig. 4i).

Overall, our ultrastructural analyses indicate that solely expressing DA1 or DA2B myosin yields serious myofibril assembly defects, with progressive structural degeneration. The *F437I* DA1 allele results in less severe deterioration than the *A234T* DA2B allele*.* For heterozygous organisms, a state more analogous to that of patients, defects are less severe. We observed normal myofibril assembly in *F437I*/+ organisms, with very minor structural defects in adults. This contrasts with both assembly and stability defects observed for *A234T*/+ IFMs. Again, the severity of the defects seen in the *Drosophila* models mimics the differential severity of the DA1 and DA2B human syndromes.

### DA1 *F437I* heterozygote muscle fibers display reduced power output, enhanced stiffness, and depressed ATP affinity

**Fig. 5:**
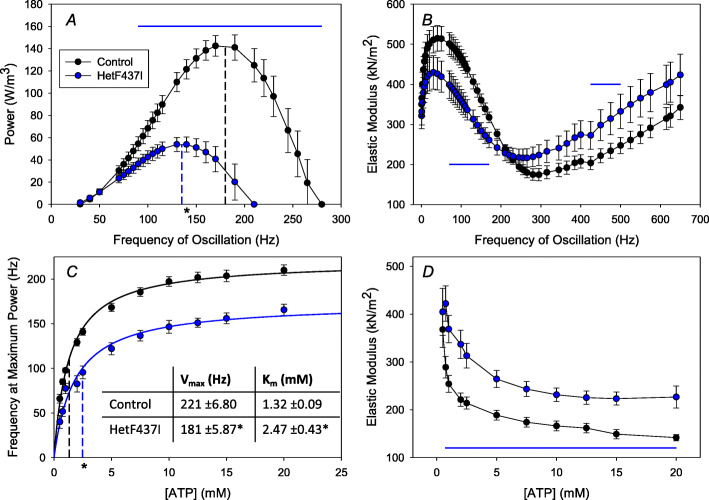
Mechanical analysis of IFMs from *F437I* heterozygotes (HetF437I). **a** HetF437I fibers generate 59% less power than the control and generate less power at frequencies greater than 90 Hz (horizontal line, *P* < 0.05, Student’s *t* test). HetF437I fibers also generate maximum power at a lower oscillation frequency, indicated by the vertical dashed lines (*P < 0.05, Student’s *t* test). **b** Elastic modulus is significantly decreased in the HetF437I fibers between 70 and 170 Hz (*P* < 0.05), but shows a trend toward increased stiffness between 400 and 550 Hz as indicated by the horizontal lines (*P* < 0.08, Student’s *t* test). **c** The frequency at which maximum power was generated (*f*_*max*_) was significantly lower in HetF437I mutant fibers at all ATP concentrations tested. *f*_*max*_ versus [ATP] data fit with the Michaelis-Menten equation showed that HetF437I fibers have a significantly (*P* < 0.001) lower *V*_*max*_ and a significantly (*P* < 0.05) higher *K*_*m*_ (table, inset). A higher *K*_*m*_ value suggests F437I myosin has a lower affinity for ATP. **d** Elastic modulus is significantly (*P* < 0.01) higher at ATP concentrations of 0.75 to 20 mM. *n* = 13 and 12 for control and HetF437I in *A* and *B*, 11 and 10 for control and F437I in *C*, and 10 and 9 for control and F437I in *D*

We wished to gain an understanding of the mechanical defects imparted by DA mutations to help define the mechanistic basis of the disease. For the genotypes studied here, only *F437I* heterozygote IFM fibers are amenable to muscle mechanical analysis, as they displayed essentially normal myofibrillar structure (Fig. 4d–f). We therefore performed sinusoidal analysis on 2-day-old *F437I* heterozygote and control fibers, which allowed us to assess the effects on fiber power production. We found that mutant fibers showed a 59% reduction in maximum power (*P*_*max*_) generated (63 ± 7 W/m^3^) compared to the control value (154 ± 9 W/m^3^) (Table 2, Fig. 5a). The frequency of maximum power generation (*f*_*max*_) in the heterozygous mutant was 146 ± 7 Hz compared to 184 ± 7 Hz in control fibers (Table 2, Fig. 5a, dashed lines), demonstrating that the DA1 allele slowed muscle fiber kinetics by 20%.

**Table 2:** Mechanical properties of IFM fibers from 2-day-old control and *F437I* heterozygote female flies

Line name (*n*)	*P*_*max*_ (W/m^3^)	*f*_*max*_ (Hz)	E_e_ at 500 Hz (kN/m^2^)	E_f_ (Hz)	Isometric Tension (mN/mm^2^)	2πb (s^-1^)	2πc (s^-1^)
*pwMhc2*/+^*pwMhc2*^ (13)	154 ± 9	184 ± 7	247 ± 20	279 ± 7	2.89 ± 0.22	1625 ± 89	2866 ± 129
*F437I* + ^*pwMhc2*^ (12)	63 ± 7*	146 ± 7*	332 ± 43	236 ± 12*	4.43 ± 0.56*	1259 ± 109*	3276 ± 111*

The mechanical properties ± standard errors of the mean of isolated mutant heterozygote and wild-type control fibers were assessed. Maximum power (*P*_*max*_), frequency where maximum power was generated (*f*_*max*_), the frequency at lowest elastic modulus (E_f_), isometric tension, and muscle apparent rate constants 2πb and 2πc were all significantly different for *F437* heterozygote fibers compared to control fibers (* = *P* < 0.05, Student’s *t* test). Values are mean ± standard errors of the mean

The slowed muscle kinetics were due to alterations in at least two rate constants of the cross-bridge cycle, based upon changes to muscle apparent rate constants 2πb and 2πc. Deconvoluting the complex modulus obtained from sinusoidal analysis into its work-producing and work-absorbing components revealed that the rate constant for work production, 2πb (which is primarily influenced by actin attachment, Pi release, and the power stroke), was decreased by 23% and the rate constant for work absorption, 2πc (primarily influenced by steps associated with cross-bridge detachment such as ADP binding and ATP-induced detachment), was increased by 14% (Table 2).

Increased muscle stiffness has been reported as a phenotype of DA [1]. Thus, we measured passive and active stiffness of the *F437I* heterozygote and control fibers by assessing elastic modulus values at 50 different frequencies, ranging from 0.5 to 650 Hz [23]. There was no significant difference in passive (pCa 8.0) elastic modulus (muscle stiffness) between the mutant and control fibers, with values of 370 ± 35 kN/mm^2^ compared to 352 ± 25 kN/mm^2^ at 500 Hz, respectively (*P* = 0.43, Student’s *t* test). The activated fibers showed values suggesting increased elastic modulus between 400 and 500 Hz compared to the control, although this was not statistically significant (*P* = 0.0775) (Fig. 5b and Table 2). An increased elastic modulus value at high frequencies would suggest either an increased number of cross-bridges bound to actin and/or increased myosin stiffness. Similarly, the 35% higher isometric tension caused by the mutation (Table 2) could be explained by increases in either of these two parameters, and/or in the case of tension, increased cross-bridge working stroke distance (step size).

To further assess the consequences of the *F437I* mutation on muscle stiffness and to examine effects on the steps of the cross-bridge cycle involving ATP binding, we measured elastic modulus and *f*_*max*_ values over a range of ATP concentrations. As shown in Fig. 5c, the mutant fibers have a lower affinity for ATP, indicated by a 1.9-fold larger *K*_*m*_ value compared to control fibers (1.32 ± 0.09), suggesting that cross-bridge rates associated with ATP binding are somewhat reduced. *V*_*max*_ decreased in the mutant fibers by ~ 20%, almost exactly the same percentage as for *f*_*max*_ under optimized power conditions (Table 2). Decreased elastic modulus at 500 Hz was consistently observed when measured over a range of ATP concentrations (Fig. 5d), suggesting our earlier elastic modulus measurement (Fig. 5b and Table 2) was valid in spite of not being statistically significant at the *P* < 0.05 level. As the ATP concentration decreased, the ATP concentration versus elastic modulus slope increased more rapidly for mutant fibers than for the control (Fig. 5d). This again suggests that there is a reduced affinity for ATP by myosin in the mutant fibers, which contributes to the increased muscle stiffness.

We utilized the workloop technique to measure the power and work generated by *F437I* heterozygote and control IFM fibers at large amplitude muscle length oscillations, which are more similar to those occurring during in vivo locomotion than the shorter sinusoidal analysis length changes*.* First, we determined the optimal frequency of muscle oscillation and percent muscle length change (strain) that produced maximum power generation. The mutation caused a 48% decrease in net-work and a 61% decrease in power compared to control fibers (Table 3, top 2 rows). The control fibers produced maximum power at an oscillation frequency (*f*_*wmax*_) of 150 ± 8 Hz and 0.75 ± 0.18% muscle length (ML) change, while the *F437I* heterozygote fibers’ maximum power generating conditions were 117 ± 11 Hz and 0.75 ± 0.00% ML (Table 3, top 2 rows). Second, we compared the power and work produced by the mutant fibers under the control fibers’ optimal power-producing conditions (Table 3 bottom 2 rows). This second workloop power measurement showed an even greater loss of net-work and power, with a 64% decrease in both parameters (Table 3, bottom 2 rows). This loss of cyclical power production is likely due to a combination of slower myosin kinetics and an increase in time myosin spends bound to actin. Increased time-bound would increase resistance during the lengthening portion of the work loop cycle causing a net loss of work and hence power.

**Table 3:** Workloop analysis of IFM fibers from 2-day-old control and *F437I* heterozygote female flies

Line name (*n*)	Work (nJ/mm^3^)	Power (W/m^3^)	*f*_*wmax*_ (Hz)	%ML
*pwMhc2*/+^*pwMhc2*^ (13)	2.7 ± 0.3	392 ± 41	150 ± 8	0.75 ± 0.18
*F437I*/+^*pwMhc2*^ (12)	1.4 ± 0.3*	151 ± 25**	117 ± 11**	0.75 ± 0.00
*pwMhc2*/+^*pwMhc2*^ (12)	2.5 ± 0.2	370 ± 40	150	0.75
*F437I*/+^*pwMhc2*^ (6)	0.9 ± 0.7*	134 ± 46**	150	0.75

Workloop analysis of isolated fibers from mutant heterozygote and wild-type control fibers. For the top 2 rows, optimal muscle length oscillation frequency (*f*_*wmax*_), and % muscle length (%ML) change were varied until maximum power was generated by the fiber. In the bottom 2 rows *f*_*wmax*_ and %ML were set at the values that produced maximum power for the control line and the resulting work and power recorded. Values are mean ± standard errors of the mean. Statistically significant differences between mutant and wild-type fibers were found for work, power, and *f*_*wmax*_ (top two lines) and for work and power at 150 Hz and 0.75% ML (lower two lines) (* = *P* < 0.05, ** = *P* < 0.01, Student’s *t* test)

### DA1 F437I myosin shows reduced ATPase activity and in vitro actin filament sliding

We examined the effects of the F437I mutation at the molecular level using myosin isolated from *F437I* IFM for ATPase activity measurements and in vitro actin filament sliding velocity (Table 4)*.* The basal Mg-ATPase level was significantly reduced compared to wild-type control (0.071 ± 0.047 s^-1^ vs. 0.228 ± 0.039 s^-1^, respectively). Actin activation of the Mg-ATPase for F437I myosin was poor, yielding a dramatic reduction in *V*_*max*_ relative to wild type (0.190 ± 0.054 s^-1^ vs. 1.682 ± 0.365 s^-1^, respectively). *K*_*m*_ for actin affinity relative to ATPase activity did not differ significantly (0.562 μM ± 0.222 μM vs. 0.692 ± 0.154 μM, respectively). Overall, this led to a dramatic reduction in the catalytic efficiency, the ratio of *V*_*max*_ to *K*_*m*_, of the mutant myosin compared to control (0.429 ± 0.309 s^-1^ μM^-1^ vs. 2.506 ± 0.685 s^-1^ μM^-1^, respectively). In vitro motility assays yielded a 55% reduction in actin filament velocity for F437I myosin compared to control (3.19 ± 0.48 μm s^-1^ vs. 7.11 ± 0.72 μm s^-1^, respectively). Clearly, the functional properties of myosin were negatively affected by the F437I mutation. For *A234T*, the volume of intact thoracic muscle was dramatically reduced in the mutant, obviating the isolation of adequate amounts of myosin from dissected IFM for performing these functional tests.

**Table 4:** ATPase and in vitro motility values for DA1 (F437I) myosin

Myosin isoform (*n* for ATPase/motility)	Basal Mg-ATPase (s^-1^)	Actin-stimulated *V*_*max*_ (s^-1^)	Actin-stimulated *K*_*m*_ (μM)	Catalytic efficiency (s^-1^/μM)	Motility (μm/s)
*pwMhc2-*control (6/8)	0.228 ± 0.039	1.682 ± 0.365	0.692 ± 0.154	2.506 ± 0.685	7.11 ± 0.72
F437I-DA1 (6/7)	0.071 ± 0.047 ***	0.190 ± 0.054 ****	0.562 ± 0.222	0.429 ± 0.309 ***	3.19 ± 0.48****

For ATPase assays, two technical replicates were averaged to obtain the values for each biological replicate (*n* value). For in vitro motility, mean values of at least 30 motile filaments are included for each biological replicate (*n* value). Standard deviations are indicated for each mean. Statistical significance was determined using Student’s *t* test. Significant differences were assumed for *P* < 0.05 (*** = *P* < 0.001, **** = *P* < 0.0001)

### Molecular modeling of DA1 (F437I) and DA2B (A234T) mutant myosin predicts changes in interactions

**Fig. 6:**
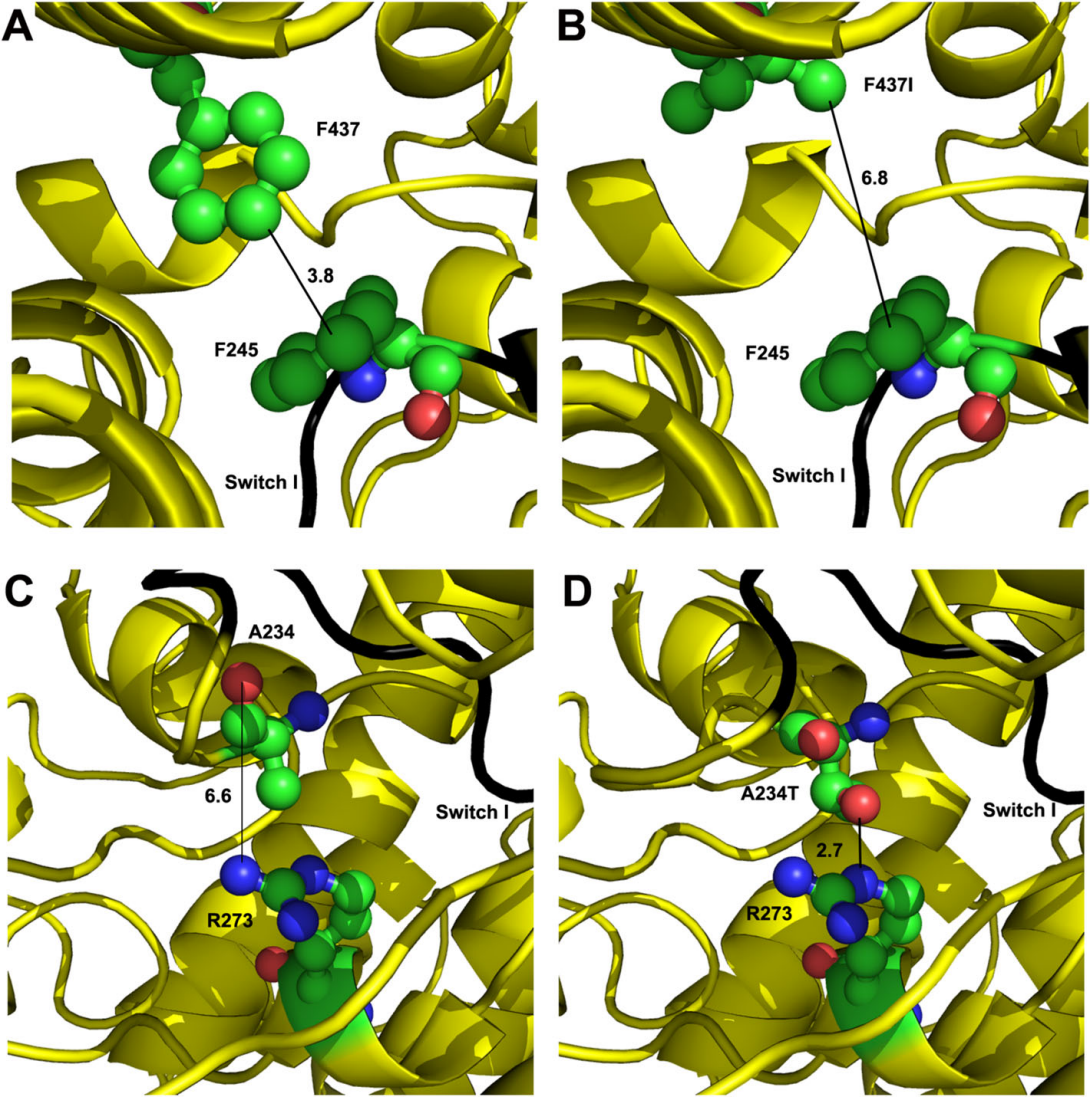
Potential amino acid residue interactions with wild-type residues A234 and F437 as well as with mutant residues A234T and F437I. The *Drosophila* IFM myosin isoform sequence was modeled onto the scallop muscle myosin II crystal structure in the pre-power stroke state (PDB 1QVI). Switch I is shown in black. Carbon, oxygen, and nitrogen atoms are shown in green, red, and blue, respectively. **a** Potential hydrophobic interaction between F437 and F245 (located on switch I), with a contact distance of 3.8 Å. **b** Disruption of the hydrophobic interaction in F437I, with the contact distance extended to 6.8 Å. **c** Location of A234 in wild type. **d** Newly formed hydrogen bond interaction between A234T and R273 (not seen in wild type), with a contact distance of 2.7 Å

Scallop muscle myosin II in the pre-power stroke state (PDB 1QVI) was used as a template to model *Drosophila* myosin in order to examine changes in molecular interactions within DA1 or DA2B mutant *Drosophila* myosins (Fig. 6). Using scallop structures for this purpose is advantageous in that crystal structures have been determined for multiple steps of the mechanochemical cycle [33–35]. We therefore also modeled interactions at the end of the mechanochemical cycle for the actin-detached post-power stroke state (PDB 1KK8). For the DA1 F437 wild-type residue located in helix O [36], a hydrophobic interaction occurs with F245 at switch 1, with a contact distance of 3.8 Å (Fig. 6a). The DA1 mutant residue, F437I, is unable to form this hydrophobic interaction and the contact distance for the mutant residue is increased to 6.8 Å (Fig. 6b). This likely disrupts a communication pathway between the nucleotide-sensing function of switch 1 and helix O in the upper 50 kD domain (Fig. 6b), an interaction required for actin release upon ATP binding [30]. In the post-power stroke state, the F437I mutation destroys the hydrophobic interaction and increases the contact distance from 3.6 Å to 4.6 Å (not shown). Molecular modeling of wild-type myosin in the pre-power stroke state (Fig. 6c) and comparison to A234T myosin (Fig. 6d) indicates the formation of a new hydrogen bond between the mutant residue and positively charged R273, with a contact distance of 2.7 Å. This could hinder conformational changes necessary for progression through the mechanochemical cycle, specifically movement of adjacent switch 1 (residues 238-246), again potentially disrupting actin release. A similar new interaction (2.9 Å) between these residues occurs in the post-power stroke state (PDB 1KK8) for the A234T mutant myosin (not shown). No additional changed interactions were observed for either DA1 or DA2B residues.

## Discussion

### Disparate functional performance in *Drosophila* models of DA1 and DA2B and genotype-phenotype relationships in human DA

We built and studied the first models of human distal arthrogryposis type 1 and type 2B by combining the powerful genetic tools available in *Drosophila melanogaster* with the ability to perform an integrative analysis of muscle structure and function for these myosin-based syndromes. Our models reflect the disparate phenotypes of the two human disorders and yield insights into the myofibrillar and locomotory defects engendered by the mutations. Further, through fiber mechanical studies, myosin biochemical assays and molecular modeling, we were able to gain a mechanistic understanding of how the specific mutations yield abnormal phenotypes.

Determining the mechanism by which *MYH3* mutations lead to DA1 and DA2B phenotypes has been difficult, due to the lack of disease models as well as the paucity of patient muscle samples. In contrast, our *Drosophila* models allowed expression of the mutant alleles in the IFM and jump muscles, in the absence of wild-type myosin, offering the opportunity to define the functional, structural, and biochemical effects of a pure population of DA mutant myosin. By expressing one mutant and one wild-type copy of *Mhc* in these muscles, we were able to assess the dominant defects engendered in vivo and in isolated DA1 IFM fibers. This heterozygous condition more closely mimics the clinical situation for DA, since patients typically have only one copy of the dominant mutant allele.

Our results convincingly demonstrated that DA1 (*F437I*) transgenic flies display less severe functional defects than DA2B (*A234T*) organisms, which is consistent with human DA classification criteria [4]. Notably, *F437I* homozygotes showed dramatically longer lifespans compared to *A234T* homozygotes (Fig. 2a). Further, while *F437I* mutants were flightless only as homozygotes, *A234T* mutants were flightless as both homozygotes and heterozygotes (Table 1). *F437I* heterozygotes, however, showed a somewhat reduced flight index and wing-beat frequency compared to control values, illustrating the dominant nature of the mutation. Similar functional disparities were observed for jump muscle, with DA1 flies displaying jump capabilities that were moderately reduced compared to control homozygotes and heterozygotes. In contrast, the DA2B mutation nearly disabled jumping in both homozygotes and heterozygotes.

In comparison to these clear phenotypic disparities between our *Drosophila* DA models, the correlation between human DA genotype and phenotype is not always consistent. For instance, the *F437I* DA1 allele studied here showed variable penetrance within members of a multi-generational family, with differential extremity contractures and various ages of disease onset [28]. Further, our modeled DA2B allele *A234T* [8] was subsequently observed in DA1 patients [37]. Other cases of phenotypic variability exist. *T178I* was initially classed as a mutation shared between DA2A and DA2B [3], but it was reclassified as a DA2A mutation [38]. Also, patients with the *R672H* DA2A mutation can show different degrees of limb and facial contractures [3]. Overall, there is a low genotype-phenotype correlation based on patients’ clinical features [39], as there can be phenotypic variability of a given allele within the same DA classification and some alleles can be classed into more than one DA category. Likely, modifier genes, epigenetic influences, and environmental factors play a role in the phenotypic variability observed. The *Drosophila* model system controls for these variables, leading to more straightforward conclusions about direct genotype-phenotype relationships.

### Distinct structural defects in DA1 and DA2B transgenic flies

Our electron microscopy analyses showed that the IFM structure of both *F437I* and *A234T* homozygotes was severely disrupted as early as the late pupal stage, indicating that these myosin head mutations appear to affect myofibril assembly and stability, with the more severe DA2B mutation showing greater disruption (Fig. 3). Although the myosin rod domain is typically considered the key factor in thick filament and myofibril assembly, previous studies have shown that mutations in the myosin head domain can disrupt myofibrillogenesis [13, 14, 27]. The ultrastructural disparity induced by the *F437I* and *A234T* alleles was even more stark in heterozygotes, as assembly and stability of myofibrils were nearly equivalent to wild type for *F437I* heterozygotes, whereas severe myofibrillar defects occur in late pupal-stage *A234T* heterozygote IFMs (Fig. 4g). *A234T* heterozygote muscles subsequently degenerate and this may be linked with hypercontraction (Fig. 4h,i). Again, the relative severities of the human syndromes are mirrored in the *Drosophila* models. Unexpectedly, however, three of the DA2A alleles that we previously studied [13] showed ultrastructural phenotypes that were intermediate between the type 1A and type 2B alleles examined here, rather than displaying even more severe defects, as might be expected based upon the human condition. As heterozygotes, all three DA2A alleles displayed normal myofibril assembly, which was followed by degeneration in young adults. Thus, in the *Drosophila* model, the *A234T* DA2B allele is a particularly penetrant and deleterious mutation.

Few studies on human DA muscle ultrastructure have been reported, and those in the literature were limited to analysis via light microscopy. Kimber and colleagues [39] described histological staining results of muscle biopsy specimens from DA patients and noted a few pathological changes, although the specific muscle types examined were not described. Two DA2B patients with *MYH3* mutations displayed variable fiber sizes, including a high degree of small type-1 fibers. Further, a DA2A patient with a *MYH3* mutation showed a predominance of type-1 fibers or scattered small type-1 fibers. In contrast to these observations, Racca et al. reported that DA2A patient gastrocnemius muscle displayed “relatively normal muscle architecture” upon histological analysis [10]. They did not observe changes in fiber type as a result of the disease. However, they reported an increase in central nuclei, suggesting possible necrosis and muscle regeneration. Interestingly, while Portillo et al. discerned normal tissue architecture in a biopsy of a DA2A patient’s right vastus medialis, they observed fibrous and adipose tissues with no skeletal muscle in biopsies of the obicularis oculi [40].

Although there are only limited human histological results, the *Drosophila* models do show some similarities to the human disease states and may yield new insights. Certainly, the lack of ultrastructural defects in heterozygotes of our DA1 model is similar to some of the human disease reports, whereas the severe phenotype seen in our DA2B heterozygote, which leads to muscle degeneration, might account for the fibrous and adipose tissue replacement observed for one human patient. In making such comparisons, it is important to note that the *Drosophila* heterozygote models likely yield equimolar levels of wild-type and mutant protein, whereas human muscle tissues standardly display changes in isoform levels during development and are capable of disease-based compensatory changes in isoform expression due to the presence of multiple myosin genes [11]. The *Drosophila* models can still serve as beneficial tools to simplify our understanding of disease development and to establish the correlation between locations of mutations in the myosin molecule and severities of syndromes.

### Mechanical, biochemical, and molecular modeling studies yield insight into the mechanism of DA

The DA1-causing *F437I* myosin mutation in its heterozygous state resulted in significant alterations in contractile properties of *Drosophila* IFM fibers. This mutation increased active fiber stiffness and decreased *f*_*max*_, power, work generation, and ATP affinity (Fig. 5; Tables 2 and 3). These results suggest that human DA1 muscle contractures form due to slowed cross-bridge kinetics and decreased muscle power generation. A decrease in ATP affinity would contribute to an increase in time myosin spends bound to actin by hindering the detachment step of the cross-bridge cycle. Prolonged binding would account for the increased muscle stiffness and contribute to decreased cyclical power production. The decreased *f*_*max*_ and power generation effectively impair flight ability, as shown by the decreased wing beat frequency in the mutants. These results for DA1 agree with conclusions regarding *Drosophila* models of two DA2A heterozygotes (*Y583S* and *T178I*), where increased stiffness, decreased *f*_*max*_, power, and work generation contribute to an increase in duty ratio, the fraction of time the myosin head is attached to actin during the mechanochemical cycle [41]. Further, a slower relaxation rate observed in *Drosophila* jump muscle for these two DA2A myosins (manuscript in preparation), and the slowed myofibril relaxation rate observed in human biopsies from *R672C*/+ DA2A patients [10] both support prolonged myosin binding to actin.

Our in vitro motility and ATPase assays are concordant with the mechanical studies suggesting slowed myosin kinetics. The ~ 50% reduction in actin sliding velocity that we observed for DA1 myosin (Table 4), and similar reductions we previously reported for our DA2A myosin models [13], support slowed cross-bridge kinetics in both classes of DA. The observed severe reductions in basal and actin activated ATPase activities (Table 4) are consistent with the slowed cross-bridge kinetics and decreased ATP affinity observed in our current muscle mechanics studies. Paradoxically, the DA1 ATPase rate reductions are more severe than those we previously observed for three DA2A myosin mutants, which showed reduction for basal Mg-ATPase for one of three lines and reductions of *V*_*max*_ for two [13]. In this regard, significant reductions in actin-activated *V*_*max*_ were observed for in vitro expressed human myosin S1 containing each of three DA2A mutant myosins [12]. Further, transient kinetic analyses of these human proteins documented reduced ATP binding for these human S1 fragments, supporting our observation for DA1 myosin in the mechanic studies (Fig. 5c). However, it is not clear that this reduced affinity would be significant at physiological ATP levels [12]. While these investigators observed reduced ATPase rates and slower detachment of the actomyosin complex, they concluded that there is likely a reduced duty ratio for the mutant proteins, as their measurements suggested that mutant myosins spend a larger proportion of the cross-bridge cycle in a detached state. The increased duty ratio we are postulating based on data from our *Drosophila* models may therefore be dependent upon the presence of an organized sarcomere where stress and strain effects play a role in setting cross-bridge kinetics.

A comparison of the molecular interactions of the DA and wild-type proteins (Fig. 6) allows a unifying hypothesis as to the mechanism by which the DA1 and DA2B mutant alleles studied affect myosin function. Both mutations appear to impair communication between switch 1 and the actin-binding site, which normally occurs via twisting of the central 7-stranded beta-sheet region of the molecule [29, 30]. Switch 1 and the P-loop move toward each other to facilitate ATP binding during this process, which also creates a cleft at the actin-binding site and releases myosin from actin. The failure of the mutant myosins to properly communicate nucleotide binding to the actomyosin interface would slow actin release, yielding decreased cross-bridge kinetics, increased stiffness, slower actin sliding, and reduced cross-bridge cycling that we observed in the muscle mechanics, in vitro motility, and ATPase activity assays. It is further possible that impaired ADP release due to the abnormal nucleotide pocket conformation could contribute to slowing myosin detachment from actin.

## Conclusions

We have produced and analyzed the first models for DA1 and DA2B, which have provided insights into the mechanism of myosin-based distal arthrogryposis. The differential severity of the human diseases is illustrated by disparate longevity, flight and jump muscle function, and myofibrillar structure in the *Drosophila* models. Further, our mechanical, in vitro motility, and ATPase results for the DA1 model suggest that slower cross-bridge kinetics and particularly a longer spent time of myosin bound to actin causes the increased muscle stiffness that mirrors the human contracture phenotype. The *Drosophila* models obviate the variability often observed in DA patients with the same *MYH3* mutations, which is likely influenced by modifier genes, living environments, and physical treatments. This, coupled with the ability to perform an integrative analysis of mutation effects from the level of the isolated protein through ultrastructural and physiological consequences, should continue to allow the *Drosophila* models to yield important insights into the disease process and possible therapies. For example, our studies suggest that 2-deoxy-ATP, which facilitates cross-bridge detachment and increases cross-bridge kinetics [42–44], might be investigated as a useful therapeutic candidate for DA patients.

## Data Availability

The DNA constructs, fly lines, and any primary data not included in the manuscript are available from the corresponding author upon reasonable request.

## Abbreviations

DA: Distal arthrogryposis
E_e_: Elastic modulus
E_f_: Frequency at the lowest elastic modulus
*f*_*max*_: Frequency where maximum power was generated
*f*_*wmax*_: Frequency where maximum power was generated in workloop assays
IFM: Indirect flight muscle
*Mhc*: Myosin heavy chain gene (*Drosophila*)
ML: Muscle length
*MYH3*: Myosin heavy chain 3 gene (human embryonic)
*P*_*max*_: Maximum power
